# Patents for New Drugs

**Published:** 1915-08

**Authors:** 


**Patents for New Drugs.** P. Gallois, Bull. et Mem. de la Soc. de Med. de Paris, May 23, discusses the dependence of various countries, France especially, on German products and advocates the adoption of a resolution by the Society, to the effect that the formidable development of the chemic industries in Germany have been due to the issuance of patents to physicians and that therapeutic discovery and available new drugs would be fostered by similar measures in France. No definite action seems to have been taken.

